# Explicit Context Matching in Content-Based Publish/Subscribe Systems

**DOI:** 10.3390/s130302945

**Published:** 2013-03-01

**Authors:** Sergio Vavassori, Javier Soriano, David Lizcano, Miguel Jiménez

**Affiliations:** 1 School of Computer Science, Universidad Politécnica de Madrid, 28660-Boadilla del Monte, Madrid, Spain; E-Mails: svavassori@conwet.com (S.V.); jsoriano@fi.upm.es (J.S.); mjimenez@conwet.com (M.J.); 2 School of Computer Science, Open University of Madrid (UDIMA), 28400-Collado Villalba, Madrid, Spain

**Keywords:** content-based publish/subscribe, context-awareness, contextual scoping, overlay network

## Abstract

Although context could be exploited to improve performance, elasticity and adaptation in most distributed systems that adopt the publish/subscribe (P/S) communication model, only a few researchers have focused on the area of context-aware matching in P/S systems and have explored its implications in domains with highly dynamic context like wireless sensor networks (WSNs) and IoT-enabled applications. Most adopted P/S models are context agnostic or do not differentiate context from the other application data. In this article, we present a novel context-aware P/S model. SilboPS manages context explicitly, focusing on the minimization of network overhead in domains with recurrent context changes related, for example, to mobile ad hoc networks (MANETs). Our approach represents a solution that helps to efficiently share and use sensor data coming from ubiquitous WSNs across a plethora of applications intent on using these data to build context awareness. Specifically, we empirically demonstrate that decoupling a subscription from the changing context in which it is produced and leveraging contextual scoping in the filtering process notably reduces (un)subscription cost per node, while improving the global performance/throughput of the network of brokers without altering the cost of SIENA-like topology changes.

## 1. Introduction

Despite potentially featuring large numbers of nodes, most sensor networks currently work as isolated islands, and most of the sensed valuable data is not yet shared. In the last few years, some attempts have been made to share data with the global community, but they focus on sharing across multiple wireless sensor networks (WSNs), spatially deployed in different locations (*i.e.*, bridging WSNs on the Internet). Indeed, most of this research comes under the Large-Scale Wireless Sensor Networks umbrella [1]; it continues to address application-specific, static-sensor deployments, and does not tackle sensed-data sharing across different applications that could exploit the data outside the sensor network. Unless sensed data are shared across different application domains, the most important feature of ubiquitous computing—namely context awareness—will not easily reach its full potential.

With the advent of the Internet of Things (IoT), we can envision ubiquitous next-generation sensor networks that are connected to the Internet for publishing, sharing and searching sensed data across a wider range of IoT-enabled applications. The availability of advanced middleware functionalities to gather, process, exchange and exploit such data on a massive scale will be the cornerstone of the development of smart (customized, personalized and enriched) context-aware applications and services outperforming any available on the current Internet [2], thus fostering the creation of new business models and opportunities.

There is therefore an urgent need for a comprehensive solution that helps to share and use sensed data coming from ubiquitous WSNs across a plethora of applications intent on using these data to build context awareness. IoT-enabled cloud infrastructures, which are evolving to support ubiquitous and context-aware computation and information integration, could then leverage valuable sensed data to enable distributed applications to take into account the situation and context in which the information is produced or consumed. For example, services providing data could restrict the dissemination of their outputs to certain consumers based on their context, and any entity could subscribe to information whose provider matches a contextual scope.

The publish/subscribe (P/S) model in general, and its content-based form in particular, is poised as one of the most plausible approaches for achieving this goal, insofar as, traditionally, it has been the communication paradigm of choice for most WSNs. Moreover, the publisher and/or subscriber context (e.g., location information, environmental data, operating data, user preferences, *etc.*) could, if available, be relevant metadata for the routing process in a large fraction of the application domains in which the P/S model has been adopted as a smart solution for spreading information across a sizeable group of users or applications. This means that context has to be added to the P/S model and shared by publishers and subscribers.

In the IoT-enabled Internet, both generic data and context elements (*i.e.*, sensed data) are available for consumption through the same P/S interface. This will require a novel approach to the P/S model that explicitly deals with sensed data (*i.e.*, context) as a separate, first-class component to coherently offer context-awareness. In short, context has to be natively added to the classic P/S model, *i.e.*, considered explicitly in the routing and forwarding processes and shared by publishers and subscribers. Unfortunately, despite many promising proposals [3–5], a powerful, fully-fledged context-aware P/S model is yet to be created. Precisely, the research community has predominantly focused on the wireless sensor network (WSN) field, where sensed data can arguably be regarded as context [6,7], but none of the most widely adopted content-based publish/subscribe (CBPS) middleware coherently offers context-awareness, as we will see in the analysis of current solutions. For example, it is not unusual to find context merely encoded into published notifications and subscriptions and handled as generic data, an approach which, as we argue in this paper, leads to major inefficiencies. Context-awareness radically changes the way both routing and matching needs to be performed in CBPS middleware to achieve efficiency. There are two orthogonal aspects in CBPS: *matching and routing*. *Matching* matches messages minimizing a metric, usually time, whereas routing routes the messages to other network elements, where the metrics are distance and energy [8–10].

In this article, we present a novel context-aware CBPS model, SilboPS, in which the context is managed explicitly. The focus is on the minimization of network overhead by improving the matching algorithm in domains with recurrent context changes related, for example, to WSNs, MANETs and IoT-enabled applications. Examples of contexts that have high or varying, “bursty” update rates include inventorying, stock portfolios, people or vehicle locators and proximity networks. We then evaluate our solution and compare it to SIENA [11,12], which is generally considered the reference implementation for a scalable CBPS service with a relatively low reconfiguration cost [13,14], and designed to maximize both expressiveness and scalability. Specifically, we empirically demonstrate the following:

Decoupling a subscription from the changing context in which it is produced and leveraging contextual scoping in the filtering and routing processes in CA-CBPS systems notably reduces (un)subscription cost per node, while improving the global performance/throughput of the network of brokers without altering the cost of SIENA-like topology changes.

Section 2, in particular, reviews the solutions and proposals of context-aware P/S models. Section 3 presents a use case highlighting the shortcomings of current approaches. In Section 4, we describe a set of design principles conceived to deal with the above issues. Section 5 further details our design decisions for implementing the proposed design principles. Next, Section 6 explains how our model behaves in comparison with SIENA in the proposed scenario and provide evidences supporting the affirmation stated above. Finally, in Sections 7 and 8, we conclude our proposal, and present future work in this area.

## 2. Background

As we are exploring how to exploit context to improve P/S systems as common mechanism for sensor networks, this section will briefly introduce key aspects of context management and, especially, context-aware CBPS systems.

Context-aware models are usually defined by how they react to context changes: *passively or actively*[15]. When passive models learn about a context change, they simply store the context or prompt the user before applying any change, whereas active models manage changes without user interaction, enabling automatic contextual reconfiguration. Another way of classifying these models is by how a context-aware application realizes the context has changed, *i.e.*, either by *sensing* the environment or by *being notified*. Notification has the advantage of reducing communication overhead but at the risk of missing changes.

Syntactically, context can be transmitted using different message structures and syntaxes. The most widespread structures and syntaxes are in increasing order of complexity [2]: key-value pairs, markup scheme models, graphical models, object-oriented models, logic-based models, and ontology-based models. The flexibility/meaning trade-off differs from one representation to another. Key-value pairs are the best option for integration with CBPS systems since it is their canonical representation and, at the same time, has a rich internal representation for context modeling.

To deal with context [16] in CBPS systems, context information has to be processed from the viewpoints of both the subscriber and the publisher. Subscriber contextual information provides for filtering according to user location, device, preferences, *etc.* in order to get relevant, useful and appropriate information within the context. Publisher contextual information provides for message adaptation according to location or other context factors to get relevant, useful and appropriate information. Routing algorithms should bridge the gap, while leveraging both context scopes to reduce network overhead.

Context-awareness is a recent research line in the CBPS field, where most proposals are simply context agnostic. Within the context-aware approaches, most of the work has focused on implicitly context-aware strategies [17], which do not differentiate the context from the rest of application data and simply piggyback context on notifications or communicate context at subscription time. The shortcomings of these approaches are discussed in Section 3.

Most recent approaches have explored explicit context representation through an extended API to set the context that the broker network uses for notification routing and topology shaping [7]. However, these approaches have context-coupled subscriptions since both context and content filters are entangled making difficult them to manage independently.

Several middleware solutions have been designed to transparently implement context management and provisioning. The core assumption in [18] is that only effective and efficient context data distribution can pave the way to the deployment of truly context-aware services. This assumption is the basis for a unified architectural model and a new taxonomy for context data distribution. Similarly, a high-level software architecture for context data management and distribution suitable for m-commerce applications is presented in [19], but there is no detailed analysis of how the matching and the routing algorithms could be improved by using such contextual information. The comparison with other architectures is based on response time instead of throughput, being the latter the focus of this article.

Regarding the consideration of context in the filtering process, a major shortcoming of existing approaches and techniques is that they are very inefficient if profiles refer to values of context entity attributes that are subject to frequent changes (context updates). Recent research proposes the use of context-aware information filters (CIF) in addition to traditional information filters routing the input stream of messages in order to manage an input stream of context updates that are relevant to the routing process because the routing rules (a.k.a. profiles) refer to values in a context data store.

For example, [20] emphasizes the idea that information filtering systems and, therefore, their corresponding indexes must be context-aware and present AGILE (Adaptive Generic Indexing with Local Escalations), a set of extensions to existing index structures for information filtering systems aimed at dynamically adapting the index structure to the frequency of context changes. The key idea of AGILE is to dynamically adjust internal structure depending on the messages received, changing scope and accuracy accordingly AGILE focuses on adaptive index structure management and applies to traditional database workloads, e.g., transaction processing and the TPC-C benchmark. Performance experiments showed that AGILE can improve the message throughput of a context-aware information filter by as much as one order of magnitude, compared with traditional approaches to implementing information filtering systems (No Index, Eager full indexing, Partial indexing, and Lazy Updates, GBU). Nevertheless, AGILE does not separate the context function from the subscription. Although this allows their matching function to outperform ours when the context changes slowly compared with the notification rate, it is a shortcoming when compared with our solution in highly changing context scenarios, as we will explain later.

Finally, systems can vary in terms of the aspects considered as context information. Most mature projects largely equate context with location management and are known as location-based services (LBS). L-ToPSS [21] is a LBS system based on a P/S middleware that adds an extra location processing module to a typical event broker to manage possible spatial events and subscriptions. The system is designed to support window queries and *N-*nearest queries. Based on L-ToPSS, Xu and Jacobsen [22] propose efficient algorithms for location constraint evaluation. CAMEL [23] is a push-based middleware construct based on a database. Like L-ToPSS, the system is designed to support window queries and N-nearest queries. Most of the research investigates how the constraints or predicates in subscriptions can be evaluated more efficiently, but little progress has been made with respect to enhancing the expressiveness of spatial subscriptions [24].

## 3. Shortcomings of Implicit Context Management

Taking into account roles, notifications and subscriptions, the problem is necessarily strongly affected by the entity context, in particular, their location. A nave application of CBPS to such a scenario would be to merge content and context in both messages and filters, making it implicitly context-aware. Although this paper focuses on the matching problem, an in-depth analysis unveils several shortcomings both related to the routing and the matching problem that are discussed in this section.

**Figure 1.**
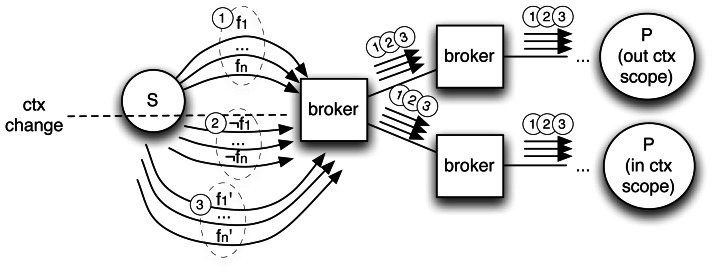
Exchange of messages with implicit context management.

- **Matching inversion.** Classic CBPS systems model messages as key-value sets, *m* ∈ { 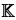 x 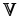 }, where 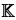 is the attribute name and 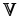 is its value. Those messages can be matched by filters issued by subscribers, *f* ∈ *F* ⊆ { 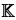 x 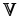 }. If *m* is in the scope of a filter *f*, we can state that *f* covers *m* or *f* ≥ *m.* The purpose of the message is to encode data, whereas the filter represents the interests in this information. Applying CBPS to context-aware scenarios violates this separation of concerns: subscriber filters contain constraints over the context mixed with constraints over the notification; symmetrically published notifications include constraints for subscribers that are enforced by brokers. As a result, there is the potential of the message and context being confused and semantically ambiguous. By mixing context and content, the application misses out on the benefits stated in [25]. In addition, this can lead to *aliasing errors* if mixed context-aware and context-unaware entities use the same P/S system.
  Only a few CBPS systems, which implement filtering using Turing-complete languages, can afford this out-of-the-box change. Anyway, such systems are difficult to optimize and exploit in the first place.
- **Message flooding in changing environments.** Published messages bundle the notification content with contextual information ( 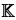 = 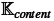 ∩ 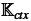), for example, the events detected by cameras include the camera location. This approach has a comparatively small overhead with respect to a context-agnostic scenario since the rate of change of the context is small compared with the rate of publication.
  If as in Figure 1 that assumption is not met, constant context updates will create a massive overhead because existing subscription filters (➀) are constantly invalidated (➁) and then updated (➂). In terms of messages, this implies a waterfall of unsubscriptions and new updated subscriptions flooding the whole network as illustrated. In our scenario, driver subscriptions pose this problem since their position changes constantly, and they are encoded as part of the filter.
- **Unrealized potential performance.** If properly exploited, contextual information can improve efficiency by minimizing networking overhead in two senses: (a) messages could be efficiently routed; (b) the overlay network topology could be optimized. For instance, it is of no use to cluster drivers interested in *nearby traffic alerts* (same interest) without considering the context that permeates their interest. Similarly, clustering drivers at nearby locations (same context) is plagued by the same problem, since it ignores their interests.
  Using implicit context, however, the broker will have to handle more complexity in the form of bigger routing tables and longer routing computation. Finally, any advantage from context exploitation requires explicit context management. Implicit context-aware solutions require the broker to parse both messages and filters, which is an additional overhead.
- **Separation of concerns within entities.** Context management and subscription or publication are very different concerns that must be handled by different components at the architectural level. Implicit context-awareness favors coupling and forces rigid designs in which the P/S component is also responsible for detecting, managing and communicating context changes.
  For instance, drivers can use a mobile application in which location context is managed by a GPS component decoupled from the subscriber component in charge of handling incoming notifications.
- **Undermined publisher/subscriber decoupling.** The main property of CBPS systems is decoupling in terms of space, time and synchronization between publisher and subscriber. The only thing that the subscriber needs to know is how the events of interest are represented. Subscribers and publishers are obliged to share a unified and homogeneous representation of context, as context is embedded in notifications for implicitly context-aware systems. In the example scenario, location is represented uniformly as a coordinate point for all entities. A more flexible approach, such as ontology-based mediation, is beyond the scope of the scheme.

## 4. Proposed Design Principles

There is a clear motivation to overcome the weaknesses of implicit context-aware management, which degrade as the application scenario context becomes more changeable.

Our approach is to decouple context and content throughout the CBPS with the aim of spatially, temporally and synchronously decoupling publishers from subscribers within a *contextual scope*.

**Figure 2.**
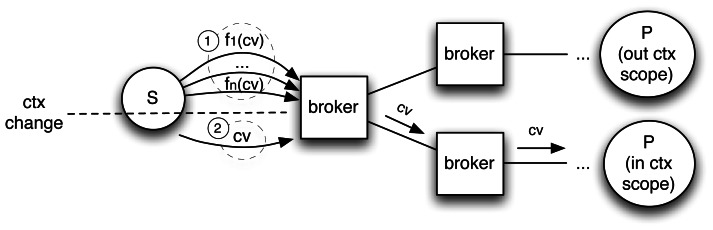
Exchange of messages with our proposed explicit context management.

The design principles that guide our vision of a context-aware content-based publish/subscribe (CA-CBPS) system are as follows:

1. *Explicit separation of context and content.* The scope of both messages and their filters must be restricted and deal exclusively with content. Therefore, *m* ∈ { 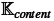 × 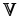 }.
2. *Generalized P/S model for contextual scoping.* The reversal of the matching procedure demonstrates an impedance mismatch between content-based filtering and any other type of filtering that does not fit the content-based model. Symmetric context-scoping filters will be attached to both subscriptions and publications, restricting publisher contexts and subscriber contexts, respectively. Such filters will be attached (**‖**) to publications, *m*‖*f_cs_*, *f_cs_* ∈ *F_ctx_*, and to subscriptions *f*‖*f_cp_*, *f_cp_* ∈ *F_ctx_* given *F_ctx_* ⊆ { 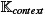 x 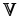}. Note how publisher and subscriber can restrict their actions symmetrically through each other's contextual scope. This scoping mechanism can be seen as a role-based access control by the publisher [26] and as a new filtering dimension by the subscriber.
3. *Context-invariant subscriptions and advertisements.* Subscriptions will be context-invariant to avoid context change flooding. Context variable references will be used instead of embedding context values (*cv*) as part of the filters. In fact, a subscription *f_s_* defined as *f*‖*f_cp_* can be regarded as a function on the context in which the filter *f_cp_* is the context-aware part:
  $$\begin{array}{c} f_{s}:\mathbb{C}\to F\\ f_{s}(ctx)\subseteq \{K\times V\} \end{array}$$
  Such functions become regular filters when applied to a given context *ctx* ∈ ℂ within the brokers. Figure 2 illustrates how flooding is avoided as subscriptions (➀) are updated by propagating context updates (➁).
  To apply context update and bind it to its correct filters, a subscriber-unique UUID, created by the broker, is added to filters and to context messages. This is necessary to distinguish the same filter if issued by different subscribers and to bind the filter to its correct context when the context function is called. This does not clash with the decoupling offered by P/S systems since it is used only internally by brokers and is comparable to the procedure enacted by other systems that use a multipath topology to resolve the duplicate message problem [27,28].

## 5. Proof of Concept: Proposed CA-CBPS Applied to the Use Case

**Figure 3.**
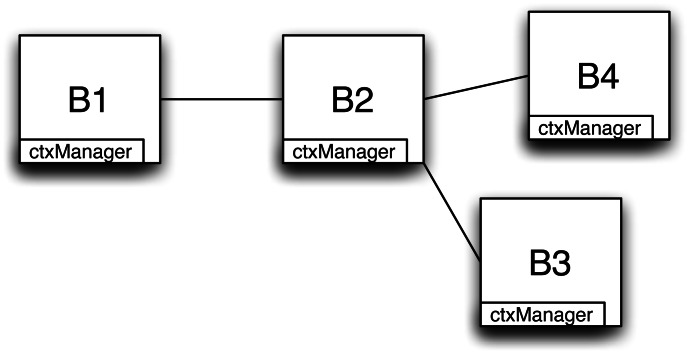
Scenario broker network.

This section shows the application of our proposal to the domain problem presented in Section 3. Our scenario includes a set of cameras sending traffic information (publishers) and a set of cars receiving relevant (context-aware) information (subscribers). Cameras are automatically connected to the brokers thanks to the infrastructure in which they are installed whereas cars can connect to brokers using the access points deployed along the road. This is an instance of a V2I scenario and is a simple sub-problem covering all the relevant details. At the same time this example fits the paper assumptions about environments with highly changeable context. Let us examine the state of the routing tables and the messages exchanged when adding publishers and subscribers to the network. Figure 3 shows the network of brokers, each of which has its own *contextManager*.

### 5.1. Adding Publishers with Changing Context

**Figure 4.**
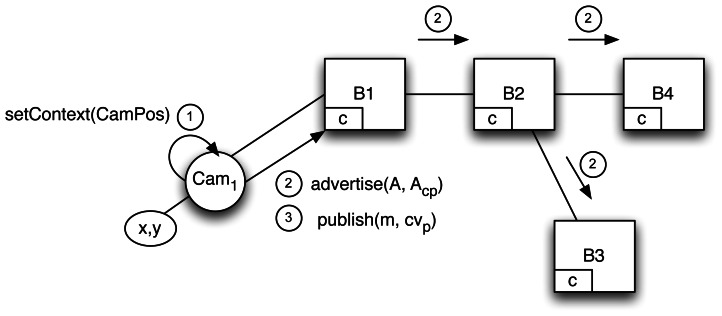
Adding a new publisher to the scenario.

A camera is connected to the broker network, specifically to broker *B_1_* whose *advertisement* and *subscription* tables are initially empty (see Figure 4). This camera establishes its initial context, namely its position, by invoking *setContext*(*CamPos*) with *CamPos* ≡ *cv_p_* = 〈*x_p_* = *CamPos.x*, *y_p_* = *CamPos.y*〉. However context updates from *Cam*_1_ will not be sent to the broker since their value is meaningful only when bound to a notification. Instead the last context will be held by the local API and appended to the sending message on each *publish* method invocation. As a matter of fact, our assumption in this scenario is that context changes are more frequent than notification/subscription messages.

*Cam*_1_ is a publisher and, as such, must send an advertisement declaring what content and context data it can provide. This information will be useful for finding out what kind of information each entity provides and what contextual variables can be used to contextualize the messages received. *Cam*_1_ sends *advertise*(*A*, *A_cp_*), where:

- *A* is an advertisement filter as in traditional CBPS systems. *Cam*_1_ can notify accidents: *A* ≡ {*msg* = *accident*}.
- *A_cp_* models the context of the publisher, *x_p_* and *y_p_*, and shows what contextual information could be exploited by subscribers to enrich their subscriptions. *Cam*_1_ has a location context: *A_cp_* ≡ {∃x_p_, ∃y_p_}.

This advertisement is propagated throughout the broker network to *B_2_*, *B_3_* and *B_4_*, producing changes in all their advertisement tables:

- *B*_1_, *advertisement* table: 〈*Cam*_1_; *A*, *A_cp_*〉
- *B*_2_, *advertisement* table: 〈*B*_1_; *A*, *A_cp_*〉
- *B*_3_, *advertisement* table: 〈*B*_2_; *A*, *A_cp_*〉
- *B*_4_, *advertisement* table: 〈*B*_2_; *A*, *A_cp_*〉

*Cam*_1_ can now send notifications to the P/S infrastructure. *B_1_* receives *publish*(*m*, *cxt*), where *m* is the notification, which is context-free content, and *cxt*, is the last context sent by *Cam*_1_. *B_1_* queries the *subscription* table to forward the message. Since it is empty, *B_1_* drops the message *m* until it receives a subscription following the path marked by previous advertisements. If the traffic camera is a mobile sensor and changes its position, it will use *setContext* to set the new position, but this will not be sent to broker *B_1_* until *publish* is invoked. Although for this scenario the context could be directly passed at publication time, interaction is cleaner if the API offers two different methods because there is no need for clients (publishers or subscribers) to remember the last context.

### 5.2. Adding Subscribers with Changing Contexts

**Figure 5.**
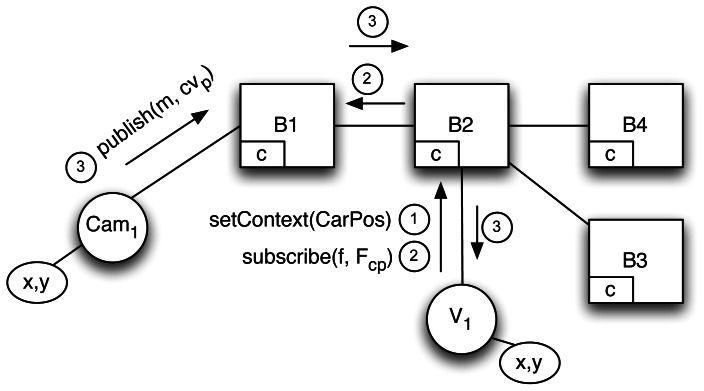
Adding a new subscriber to the scenario.

Now a first-time subscriber joins the scenario (as in Figure 5). The subscriber is a vehicle, *V_1_*, using a context-aware mobile device to receive alerts created within a 20 km radius of its position, which, for simplicity's sake, we model as a square with 40 km sides. Initially, *V*_1_′*s* location will be in the range of *Cam*_1_.

First of all, *V_1_* accesses the P/S network as a client of *B_2_* and sends its initial context, *setContext*(*CarPos*). *B_2_* receives this information as a *context message*, extracts the *cv_s_* values and creates a UUID ( $V_{1}^{id}$) for *V*_1_, which it stores to its *contextManager* table as 〈 $V_{1}^{id}$; *x_s_* = *CarPos.x, y_s_* = *CarPos.y*〉. Then *B*_2_ checks, using the *subscription* table, to see if *V_1_* context needs to be forwarded. In this case, no action is taken since the table is empty.

Next, *B_2_* receives the subscription of *V_1_* to nearby traffic alerts, *subscribe*(*f*, *f_cp_*), which is mandatory since it has sent a *setContext*(*CarPos*) call, where *f* is a content-based filter *f* ≡ {*msg* = *accident*} and *f_cp_* is a function that models a filter based on restrictions about publisher context, and receives dynamic subscriber context values:

$$\begin{array}{r} f_{cp}\equiv \{x_{p}\geq this.x-20\\ x_{p}\leq this.x+20\\ y_{p}\geq this.y-20\\ y_{p}\leq this.y+20\} \end{array}$$

The broker first adds $V_{1}^{id}$ to the new subscription *f*′, which is then stored into B_2_'s *subscription* table as 〈*V_1_*; *f′*, *f_cp_*〉. The next step is to check if there is any advertisement that matches the subscription. To do this, *B*_2_ queries its *advertisement* table, which contains row 〈*B_1_*; *A*, *A_cp_*〉 and then checks if there exists an *A* ≥ *f′* ∧ *A_cp_* ≥ *f_cp_*. Given that *A* and *f* contain *msg* = *accident* and that *A_cp_* is ∃*x_p_* ∧ ∃*y_p_* and *f_cp_* uses exactly *x_p_* and *y_p_* as the publisher context, the match is positive, and *B_2_* sends first the context message, if needed, and then the subscription message to *B*_1_. To send the context message, *B*_2_ checks whether the *subscription* table already contains an outbound entry for $V_{1}^{id}$. If it does not, it sends the context message, namely *context*(*cv_s_*) with *cv_s_* the *context value* containing 〈 $V_{1}^{id}$; *x_s_* = *CarPos.x, y_s_*= *CarPos.y*〉, to *B*_1_. Then it sends a subscription message with sub scribe′(*f*, *f_cp_*).

When *B*_1_ receives both messages, it adds the subscriber's context *cv_s_* to the *contextManager* as 〈 $V_{1}^{id}$, *cv_s_*〉 and modifies its *subscription* table with the subscriber's data, writing row 〈*B*_2_; *f′*, *f_cp_*〉. *B*_2_ and *B*_1_ then stop propagating the subscription because there are no more entries in their *advertisement* tables.

### 5.3. Notification Delivery

To illustrate how new publications are to be delivered to interested subscribers, imagine that there is an accident close to *Cam*_1_. *Cam*_1_ sends a new *publish*(*m*, *cv_p_*), where *m* ≡ {*msg* = *accident*}. The publication includes the contextual information because the previous *setContext* called by publisher did not actually send the changes to the broker.

*B*_1_'s *subscription* table contains row 〈*B*_2_; *f′*, *f_cp_*〉 and therefore *B*_1_ must check if the publish message matches these filters, dropping it otherwise. Because *m* ≥ *f*, *B*_1_ must check the contextual filter. This is a lazy process led by usually more restrictive and relevant content filters.

At this point, the context-invariant filter $f_{cp}^{\prime }$, which is the key component of our proposal, must be evaluated using the values fetched from the *contextManager* table. In particular, *B*_1_ uses $V_{1}^{id}$ that searches *f*′ to look up context information of the associated *cv′s* ≡ 〈*x_s_* =*CarPos.x′*, *y_s_* = *CarPos.y′*〉 and then passes both context (*cv_s_* and *cv_p_*) to evaluate $f_{cp}^{\prime }$:

$$\begin{array}{r} f_{cp}^{\prime }(cv_{p},cv_{s})=f_{cp}^{\prime \prime }\equiv \{\text{CamPos}.x\geq \text{CarPos}.x-20\\ \text{CamPos}.x\leq \text{CarPos}.x+20\\ \text{CamPos}.y\geq \text{CarPos}.y-20\\ \text{CamPos}.y\leq \text{CarPos}.y+20\} \end{array}$$

Note that the use of contextual values to evaluate context-aware restrictions has been delayed until now, improving performance in scenarios like this where context changes (car positions) occur at a faster rate than new notifications (traffic accidents). In addition, the context function is more expressive than the one available in subscriptions since it is able to perform simple computations and is not confined only to comparison.

In this case, the car is close enough to the camera, and the result of evaluating $f_{cp}^{\prime }$ is a positive match. Since both filters are satisfied, the message is sent to *B*_2_ as the *subscription* table prescribes. To do so, *B*_1_ generates an internal publish message *publish′*(*m*, *cv_p_*) with the contextual variables of the publisher used in the previous step, namely *cv_p_* ≡ {*x_p_* = *CamPos.x*, *y_p_* = *CamPos.y*}. Note again that the contextual data, in this case information about the publisher, is not propagated until strictly necessary.

This publication reaches *B*_2_. First, *B*_2_ accesses the *subscription* table and checks that the publication matches row 〈*V_1_*; *f′*, *f_cp_*〉. This process is identical to the above: *m* ≥ *f* so $f_{cp}^{\prime }$ must be evaluated as $f_{cp}^{\prime }$ (*cv_p_*, *cv_s_*) → $f_{cp}^{\prime \prime }$. *B*_2_ ultimately obtains the same contextual filter, and so will still match.

As the publication matches *V*_1_'s range, the message *m* carrying information about the nearby traffic incident is finally delivered to car *V*_1_.

### 5.4. Subscriber Context Update

**Figure 6.**
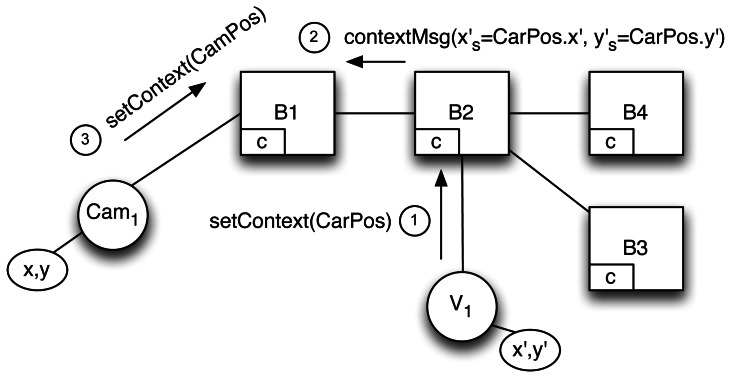
Context change of a subscriber.

First, let us consider that *V*_1_ is driving along the highway and changes its context (see Figure 6) by sending *setContext*(*CarPos*). *B*_2_ processes this change of context with the help of the context manager, which replaces the values of *x_s_* and *y_s_* previously stored in the *contextManager* table.

*B*_2_ receives the new context values *cv′s* ≡*x_s_* = *CarPos.x′*, *y_s_* = *CarPos.y*′〉, creates the $V_{1}^{id}$ and checks if it is different from the last value entered in the *contextManager* table. If it is, it updates the table with the new values, then looks up the outbound entries for $V_{1}^{id}$ used to send the context message in the *subscription* table and finds *B*_1_. If the new and old values are the same, no action is taken. Then *B*_1_ receives this context message and modifies its *contextManager* table accordingly; next it queries the *subscription* tables, repeating the process carried out by *B*_2_.

Subsequent publications by *Cam*_1_ will be subject to a new contextual match, using the new data. $f_{cp}^{\prime \prime }$ may no longer match, thus no messages will reach *B*_2_. This way, tables are updated elsewhere in the broker network for every context they receive.

### 5.5. Publisher Context Update

Now camera position changes. Again, *Cam*_1_ invokes the local API with *setContext*(*CamPos*) but no message is sent to the broker; instead the new values *cv′_p_* ≡ 〈*x_s_*= *CamPos.x′*, *y_s_* = *CamPos.y′*〉 are held locally and sent together with the following *publish* call.

## 6. Prototype Evaluation

SilboPS evolved from our Java implementation of SIENA [12] and extends its interface by adding context functionality. In this way we were able to test the two systems in the same environment and match their differences without incurring in any semantic mismatch API penalization. As usual for P/S systems, our prototype does not support transactional messages, but offers FIFO channels with reliable delivery. This implies that no message loss will be observed unless there is a link failure; however both channel endpoints are aware of such failure. Messages are processed in the same order as they arrive at broker irrespectively of their sending time.

We ran our experiment under Linux 3.2 and OpenJDK 7u3 on an Intel^®^ i7 860 @ 2.80 GHz machine equipped with 4 GB of RAM. Due to our scenario assumption, the focus is on context reconfiguration cost, rather than notification delivery performance. Nevertheless we ran a notification delivery performance test to verify the overhead introduced by this extension.

To assure that our results were meaningful, we used the same distribution parameters as in [12], adding the context part as an attribute of notifications and subscriptions for the SIENA version, and as a context function/context message in SilboPS. We used a uniform distribution applied on a square with 1,000 km sides to generate position values. The choice of this distribution gives SIENA a slight edge since it can exclude filters that do not match the notification position value, whereas SilboPS has to wait the context function result.

Thus, a context change in SIENA requires two messages to be sent: one message to remove the old filter, if any, and the second message to add the new filter. This is necessary because otherwise subscriptions will pile up leading to poor router performance and, above all, the delivery of unwanted messages.

**Figure 7.**
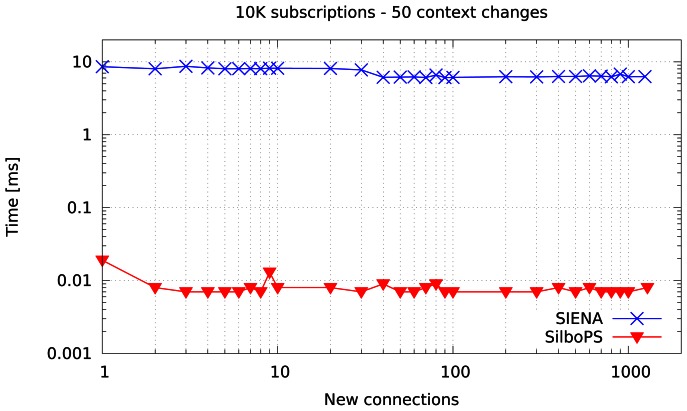
Time normalized on connection to perform 50 context change on each new subscriber.

The first experiment is meant to compare the context change cost for the two systems. It performs 50 changes per subscriber on a single broker previously loaded with 10,000 subscriptions (equivalent to 1250 connections). As shown in Figure 7, the operation has a fixed cost in both systems. However, SilboPS outperforms SIENA by three orders of magnitude. The reason is that SIENA has to modify its index entries (removing and adding the filters), instead of updating only the subscriber's context, whereas SilboPS only updates context values.

**Figure 8.**
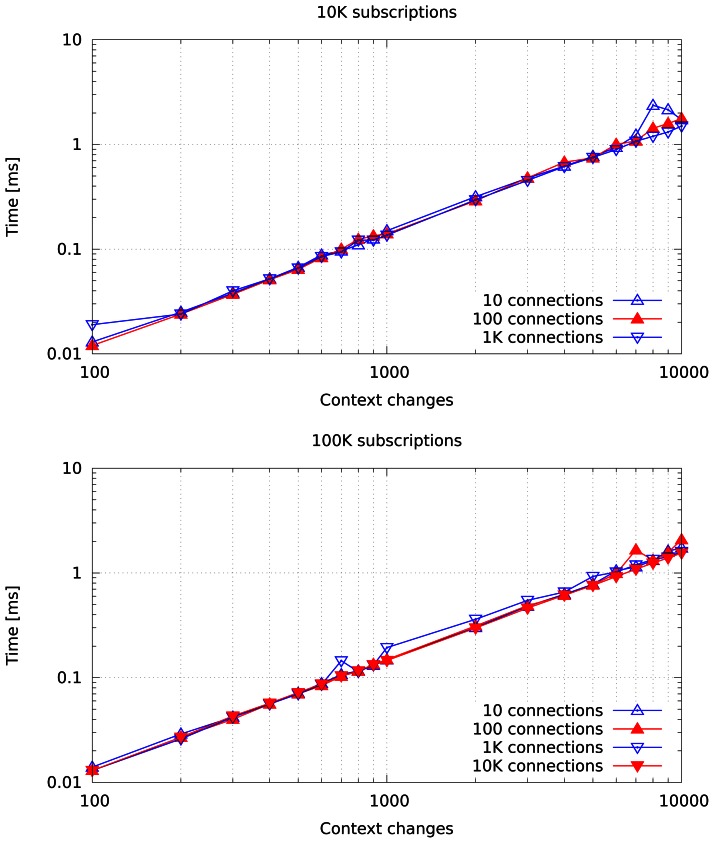
Time taken to change context with 10,000 and 100,000 subscriptions on a single broker in our prototype.

Figure 8 shows the total time taken by our prototype to perform context changes when it is loaded with an increasing number of connections. The router is loaded with a maximum of 10,000 subscriptions in the top chart and a maximum of 100,000 in the bottom chart. Each connection has an average of 10 subscriptions, that is, a load of 1,200 and 12,000 connections per router respectively. This highlights how processing time is proportional to the number of context changes and depends on neither the number of connections nor the number of subscriptions.

**Figure 9.**
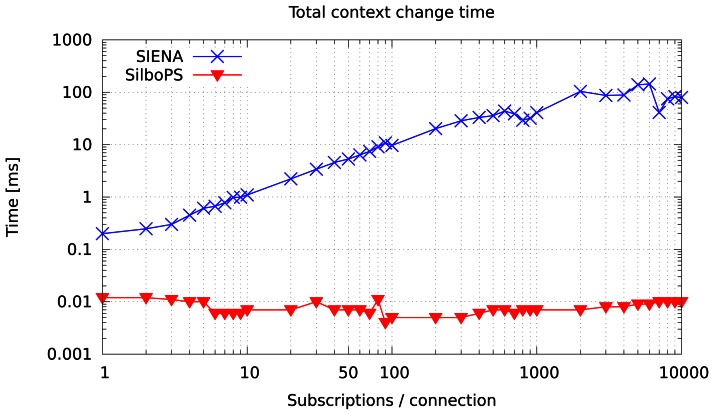
Time for a single context change with an increasing number of subscription per connection.

Figure 9 shows the cost of one context change with respect to the number of subscriptions issued by a single subscriber. It stands to reason that the cost increases linearly for SIENA, whereas it is constant in SilboPS. In addition to a faster processing time, this will reduce the number of messages sent in the network by a linear factor. So, the more subscriptions that are loaded to the broker, the bigger the saving will be. As a matter of fact, SilboPS behaves like SIENA when there is only one subscription per subscriber, but, even so, has the advantage of sending one instead of two messages, and the message size is smaller.

Note that the data in Figures 7 and 9 are consistent: the time required for a single context change in SIENA at 50 subscriptions per connection is equal to the value shown in Figure 7, that is, 10 ms. In this test SilboPS performance for a single context change appeared to be equal to its performance rate for 50 context changes. This is because, even though it has nanosecond precision, the available API does not guarantee nanosecond accuracy (As described in [29]). Even so we were able to calculate the slope for SIENA version and to get an estimate for the cost of a single context change of 105 μs/msg. For SilboPS we can either use Figure 7 where the time is high enough to be measured accurately, or use Figure 8 to calculate the slope, which is a fairly straight line. Thus we estimated an upper-bound of 0.2 μs/msg.

**Figure 10.**
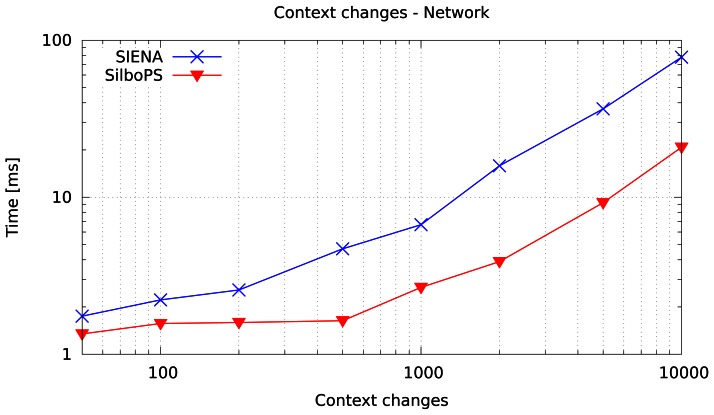
Time required for changing the context in a 5-broker network with 1 publisher and 100 subscribers each.

Figure 10 reports the time to process context change messages in a network of five brokers each with one publisher and 100 subscribers, where each subscriber sends on average 10 subscriptions for a total of 1,000 subscriptions per broker.

Our implementation is clearly about five times faster than SIENA. However, network transport protocol overhead reduces the three-order-of-magnitude gain shown earlier. Another network effect is buffering introduced by sockets. Note that the slope of the two curves changes at around 200 and 1000 context changes for SIENA and our prototype respectively; this is due to selected channel socket buffer filling. As a matter of fact, both systems use a queue to hold and then a single thread to process incoming messages in the broker. Consequently, incoming sockets do not slow down the routing since they each have their own thread. However, outgoing sockets do slow down the process because they use the broker's thread.

**Figure 11.**
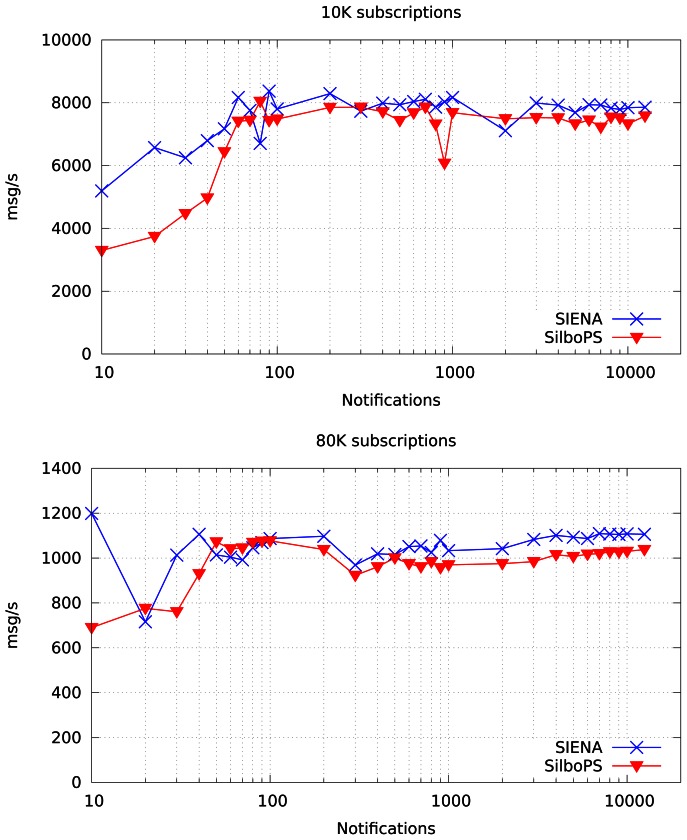
Notification throughput with 10,000 and 80,000 subscriptions in a single broker.

Figure 11 shows notification throughput when the broker is loaded with 10,000 subscriptions in the top chart, and with 80,000 subscriptions in the bottom chart. It is clear that SilboPS introduces a small overhead with respect to SIENA and that maintains the same shape through all the range; this proves our first assumption, stating that filters applied to notifications are stricter than the ones applied to context. As expected, the more notifications we send, the more stable the value is, since the broker queue always has an element and thus the broker saturation point can be reached.

These data are useful for calculating the matching time difference between the two systems. This reveals the trade-off point between the number of notifications and the number of changing subscriptions in the system, namely when it is convenient to switch from SIENA to our approach. We chose these values (see Table 1) because they represent the biggest difference between the two system's throughputs above the broker saturation point.

The trade-off point can be found by comparing the matching and reconfiguration time for the two systems. More formally:

*X_notification_* is the number of notifications
*X_context_* is the number of context changes
$T_{\text{SIENA}}^{\text{not}}$ is the average time to process a notification with SIENA, namely 915 μs/notification
$T_{\text{SIENA}}^{\text{cxt}}$ is the average time to process a context change with SIENA, namely 105 μs/context change
$T_{\text{SilboPS}}^{\text{not}}$ is the average time to process a notification with SilboPS, namely 1,000 μs/notification
$T_{\text{SilboPS}}^{\text{cxt}}$ is the average time to process a context change with SilboPS, namely 0.2 μs/context change

Thus the inequality to be applied to determine when switch to SIENA is

(1) $$X_{\text{notification}}T_{\text{SIENA}}^{\text{not}}+X_{\text{context}}T_{\text{SIENA}}^{\text{cxt}}<x_{\text{notification}}T_{\text{SilboPS}}^{\text{not}}+X_{\text{context}}T_{\text{SilboPS}}^{\text{cxt}}$$

which is solved with respect to the ratio of X's:

(2) $$\frac{X_{\text{notification}}}{X_{\text{context}}}>\frac{T_{\text{SilboPS}}^{\text{cxt}}-T_{\text{SIENA}}^{\text{cxt}}}{T_{\text{SIENA}}^{\text{not}}-T_{\text{SilboPS}}^{\text{not}}}$$

and substituting the above values, we found as trade-off point:

$$\frac{X_{\text{notification}}}{X_{\text{context}}}\approx 1.23$$

Consequently, if the ratio between *X_notification_* and *X_context_* is greater than 1.23, the SIENA processing time is better, whereas SilboPS performs better when the number of context changes is greater than the number of notifications. Note, additionally, that *X_notification_* and *X_context_* represent the *total* number, so the more subscribers in the system there are, the more likely our system is to perform better. Moreover, this supports our assumption for the scenario described in this paper, where the number of notifications (traffic issues) is lower than the number of context changes (car's position updates).

## 7. Future Work

Of the design principles presented in Section 4, we will focus now on the *generalized P/S model for contextual scoping*. According to this model, symmetric context-scoping filters are attached to both subscriptions and publications. This allows publishers to restrict notification delivery to subscribers (*i.e.*, by checking subscriber filter validity) based on both contexts. This scoping mechanism can be seen as role-based access control by the publisher [26,30] and as a new filtering dimension by the subscriber.

**Figure 12.**
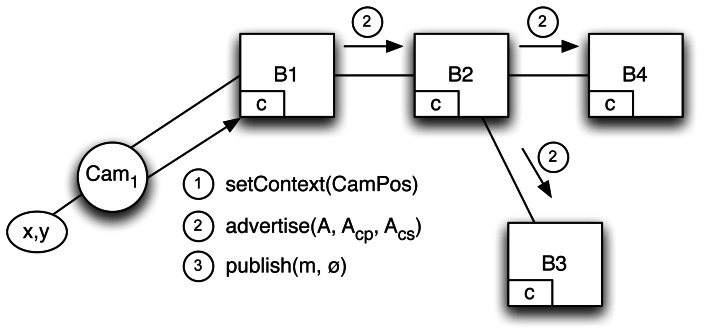
Adding a new publisher to the scenario.

This design principle will be accomplished in two stages. First, a third parameter *A_cs_* (*i.e.*, *advertise*(*A*, *A_cp_*, *A_cs_*) will be added to the advertisement *advertise*(*A*, *A_cp_*) sent by the publisher (*Cam*_1_), see Figure 12), where *A_cs_* models the context information that the publisher is going to exploit to restrict the dissemination of published messages, resembling the above role-based access control.

This advertisement is propagated through the broker network to *B*_2_, *B*_3_ and *B*_4_, leading to changes in all their advertisement tables, which now manage the publisher advertisement context for subscribers, *A_cs_*:

- *B*_1_, *advertisement* table: 〈*Cam*_1_; *A*, *A_cp_*, *A_cs_*; ∅〉
- *B*_2_*, advertisement* table: 〈*B*_1_; *A*, *A_cp_*, *A_cs_*; ∅〉
- B*_3_*, *advertisement* table: 〈*B*_2_; *A*, *A_cp_*, *A_cs_*; ∅〉
- *B*_4_*, advertisement* table: 〈*B*_2_; *A*, *A_cp_*, *A_cs_*; ∅〉

The second stage is concerned with enhancing the *publish* message, changing context values with a contextual scope filter *F_cs_* (*i.e.*, a function of the subscriber context) that publishers (*i.e.*, cameras) use to restrict the propagation of *m*, and second context values in a similar way as for subscribers with *setContext*(*CamPos*) message. As *Cam*_1_ does not restrict the scope, *F_cs_* = ∅.

This way, *Cam*_1_ can send notifications to the P/S infrastructure. *B*_1_ receives *publish*(*m*, *F_cs_*) where *m* is the notification, which is context-free content, and *F_cs_* is a contextual scope filter that the camera uses to restrict the propagation of *m*.

**Figure 13.**
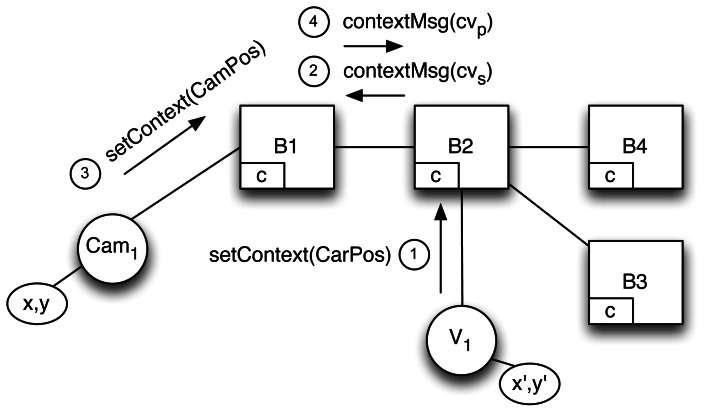
Clients change their context.

If the traffic camera is a mobile sensor and changes position, it will send new *setContext* messages to *B*_1_. The context manager of this broker will just update its local variables *x_p_* and *y_p_* with the new data and forward the message to its neighbors (see Figure 13). Further evaluation is needed regarding whether filter removal should be applied at each context change, either timed or advertisement-based.

The next steps are to evaluate the overall system performance overhead introduced by this approach, and the trade-off between this overhead and benefits for publishers that intend to take advantage of the added functionalities.

## 8. Conclusions

In this article, we have presented a novel context-aware P/S model in which the context is managed explicitly. Our model adds a new dimension to CBPS flexibility: context scoping based on context-invariant filters, *i.e.*, the notification filtering is separated from context filtering, allowing SIENA-like forwarding index to be updated only on new subscriptions and at the same time enabling a quick subscriber's context update without rebuilding the whole structure, thanks to the indirection provided by our context functions and the *contextManager* table.

Our experiments have shown that the approach presented in this article introduces a small overhead with respect to SIENA in terms of notifications throughput, but at the same time outperforms SIENA by three orders of magnitude on subscriber context updates. In addition, using our solution, clients do not have to hold the whole list of sent filters and manage filter life cycle (unsubscribe previous subscriptions, create filters with the new context and send the new subscriptions).

We have also demonstrated that our solution is extremely valuable in domains where context is highly dynamic and the number of context changes exceeds the number of notifications, such as inventorying, stock portfolios, people or vehicle locators that have with high or “bursty” context update rates.

Finally, note that the reported research is being developed and applied as part of the 4CaaSt platform-as-a-service (PaaS) project (http://4caast.morfeo-project.org), where P/S is offered as both a value-added service to hosted applications and a key internal platform asset.

## Figures and Tables

**Table 1.** Numerical result of test with 80,000 subscriptions.

Notifications	SIENA		Explicit Context		Difference	

	[msg/s]	[us/msg]	[msg/s]	[us/msg]	[msg/s]	[us/msg]
3,000	1,083	923	984	1,016	99	93
4,000	1,101	908	1,016	984	85	76
Average	1,092	915	1,000	1,000	92	85
